# *In silico* designing and immunoinformatics analysis of a novel peptide vaccine against metallo-beta-lactamase (VIM and IMP) variants

**DOI:** 10.1371/journal.pone.0275237

**Published:** 2023-07-20

**Authors:** Hamid Motamedi, Amirhoushang Alvandi, Matin Fathollahi, Marzie Mahdizade Ari, Sajad Moradi, Jale Moradi, Ramin Abiri

**Affiliations:** 1 Department of Microbiology, School of Medicine, Kermanshah University of Medical Sciences, Kermanshah, Iran; 2 Student Research Committee, School of Medicine, Kermanshah University of Medical Sciences, Kermanshah, Iran; 3 Medical Technology Research Center, Health Technology Institute, Kermanshah University of Medical Sciences, Kermanshah, Iran; 4 Department of Microbiology, School of Medicine, Iran University of Medical Sciences, Tehran, Iran; 5 Microbial Biotechnology Research Centre, Iran University of Medical Sciences, Tehran, Iran; 6 Nano Drug Delivery Research Center, Health Technology Institute, Kermanshah University of Medical Sciences, Kermanshah, Iran; 7 Fertility and Infertility Research Center, Health Technology Institute, Kermanshah University of Medical Sciences, Kermanshah, Iran; D Y Patil Deemed To Be University, INDIA

## Abstract

The rapid spread of acquired metallo-beta-lactamases (MBLs) among gram negative pathogens is becoming a global concern. Improper use of broad-spectrum antibiotics can trigger the colonization and spread of resistant strains which lead to increased mortality and significant economic loss. In the present study, diverse immunoinformatic approaches are applied to design a potential epitope-based vaccine against VIM and IMP MBLs. The amino acid sequences of VIM and IMP variants were retrieved from the GenBank database. ABCpred and BCPred online Web servers were used to analyze linear B cell epitopes, while IEDB was used to determine the dominant T cell epitopes. Sequence validation, allergenicity, toxicity and physiochemical analysis were performed using web servers. Seven sequences were identified for linear B cell dominant epitopes and 4 sequences were considered as dominant CD4^+^ T cell epitopes, and the predicted epitopes were joined by KK and GPGPG linkers. Stabilized multi-epitope protein structure was obtained using molecular dynamics simulation. Molecular docking showed that the designed vaccine exhibited sustainable and strong binding interactions with Toll-like receptor 4 (TLR4). Finally, codon adaptation and *in silico* cloning studies were performed to design an effective vaccine production strategy. Immune simulation significantly provided high levels of immunoglobulins, T helper cells, T-cytotoxic cells and INF-γ. Even though the introduced vaccine candidate demonstrates a very potent immunogenic potential, but wet-lab validation is required to further assessment of the effectiveness of this proposed vaccine candidate.

## Introduction

In recent years, two main groups of broad-spectrum beta-lactamases (β-lactamases) have emerged in gram-negative bacteria, the extended spectrum β-lactamases (ESBLs) which are more commonly found in the family of *Enterobacteriaceae*; and the metallo-β-lactamases (MBLs) or class B β-lactamases which are frequently observed in non-fermenting gram-negative bacteria [1]. The rapid spread of acquired MBLs among major gram-negative pathogens is a global concern. It’s noteworthy that the mortality rate associated with infections caused by MBLs producing organisms ranging from 18 to 67% [2]. Based on the amino acid sequence homology, MBLs belong to Ambler class B beta-lactamases and are dependent on zinc ions, and according to the Bush classification based on the substrate profiles, they belong to group 3 [3–5]. MBLs breaks the beta-lactam ring by carbapenemases activity and able to hydrolyse penicillins, cephalosporins and carbapenems [6, 7], but not monobactams [8, 9].

Today, different subclasses of B1 MBLs such as Verona integron-borne MBLs (VIM), imipenemase (IMP), Sao Paulo MBLs (SPM), Germany imipenemase (GIM) and Seoul imipenemase (SIM) have been identified [10]. Plasmid-borne MBLs, like VIM, New Delhi MBLs (NDM), IMP types may spread to different bacterial pathogens [11]. There are more than 50 different variants of VIM have been identified [12], however, structural information is provided only for VIM-2, VIM-4, VIM-7, VIM-26, and VIM-31 [13]. Currently 91 IMP variants (IMP 1–91) are reported worldwide which the first variant (IMP-1) was recognized in Japan in 1991 in *Pseudomonas aeruginosa* and *serratia marcescens* strains [14]. IMP is encoded by *blaIMP* genes on class I integron in a transferable plasmids which are easily transmitted among many gram-negative organisms [1]. IMPs are more prevalent among *Pseudomonas aeruginosa*, *Acinetobacter baumannii*, *Klebsiella pneumoniae*, *Enterobacter cloacae*, and *Escherichia coli* (*E*. *coli*) [15, 16]. IMP-producing *Enterobacteriaceae* are more prevalent in China, Japan, Iran, India, Malaysia, Singapore, Thailand, Turkey, Vietnam and Korea as well as Australia [15, 17].

MBLs are produced as protein precursors in the bacterial cytoplasm containing a signal sequence. Some of these β-lactamases such as NDM bind to lipoproteins, while VIM, SPM, and IMP present in the periplasmic space [18]. However, according to Lopez et al, similar interaction between the protein and the membrane was also observed in the case of IMP [19, 20]. Moreover, NDM and VIM enzymes are found in both soluble (V-NDM and VIM-2) and membrane lipid-binding forms (chimeric N-VIM and NDM-1) [21]. In fact, the presence of a Cys residue as lipidation cite in some variants of VIM and NDM enzyme helps them to be anchored to outer membrane. This fact is important since membrane MBLs are dispersed peripherally throughout the cell, while soluble MBLs are present only inside the cell as inclusion bodies [21]. With respect to native form of NDM [21, 22], IMP [19] and the membrane anchored variant of VIM (N-VIM) [21] these enzymes may be good targets for inactivation by the outside environment in vaccine design approach to induce effective humoral immunity [21, 23].

In recent years designing and constructing of epitope-based vaccines is increased in the pharmaceutical industry. However, epitope-based vaccines are not yet widely available in the market. It should be noted that for the first time an epitope-based vaccine against *Vibrio cholerae* and *E*. *coli* is introduced by Jacob and colleagues [24]. Only a few epitope-based vaccines against microbial pathogens have progressed to clinical trials in humans [25, 26] including Bionor Immuno’s HIV p24 gag peptide vaccine (Vacc-4X) and epitope-focused recombinant protein-based malaria vaccine (RTS,S/AS) which are in phase II and III clinical trials, respectively. Until now, these vaccines have been proven to be effective, safe and well tolerate in humans [25, 27–29].

Vaccination is an effective strategy to prevent various diseases [30]. Using bioinformatics tools, it is possible to identify conserved sequences as suitable and applicable vaccine candidates. We can use bioinformatics methods to predict the immunogenicity, allergenicity and antigenicity of candidate epitopes to elicit a reasonable and effective humoral and cellular (CD4^+^ and CD8^+^) immune responses [31–33]. Since the conserved sequences of these proteins are similar in several different bacterial genera or species, the designed vaccine candidate covers many antibiotic-resistant bacterial agents harboring IMP and/or VIM MBLs. Considering the previous study which was performed on designing *in silico* vaccine against NDM enzyme variants [22], this study intend to continue the previous study and complete data in regard to designing effective multi-epitopes vaccine against two other highest clinical MBL enzymes, VIM and IMP by immunoinformatic methods.

## Materials and methods

### Retrieval of VIM and IMP proteins

**Fig 1:**
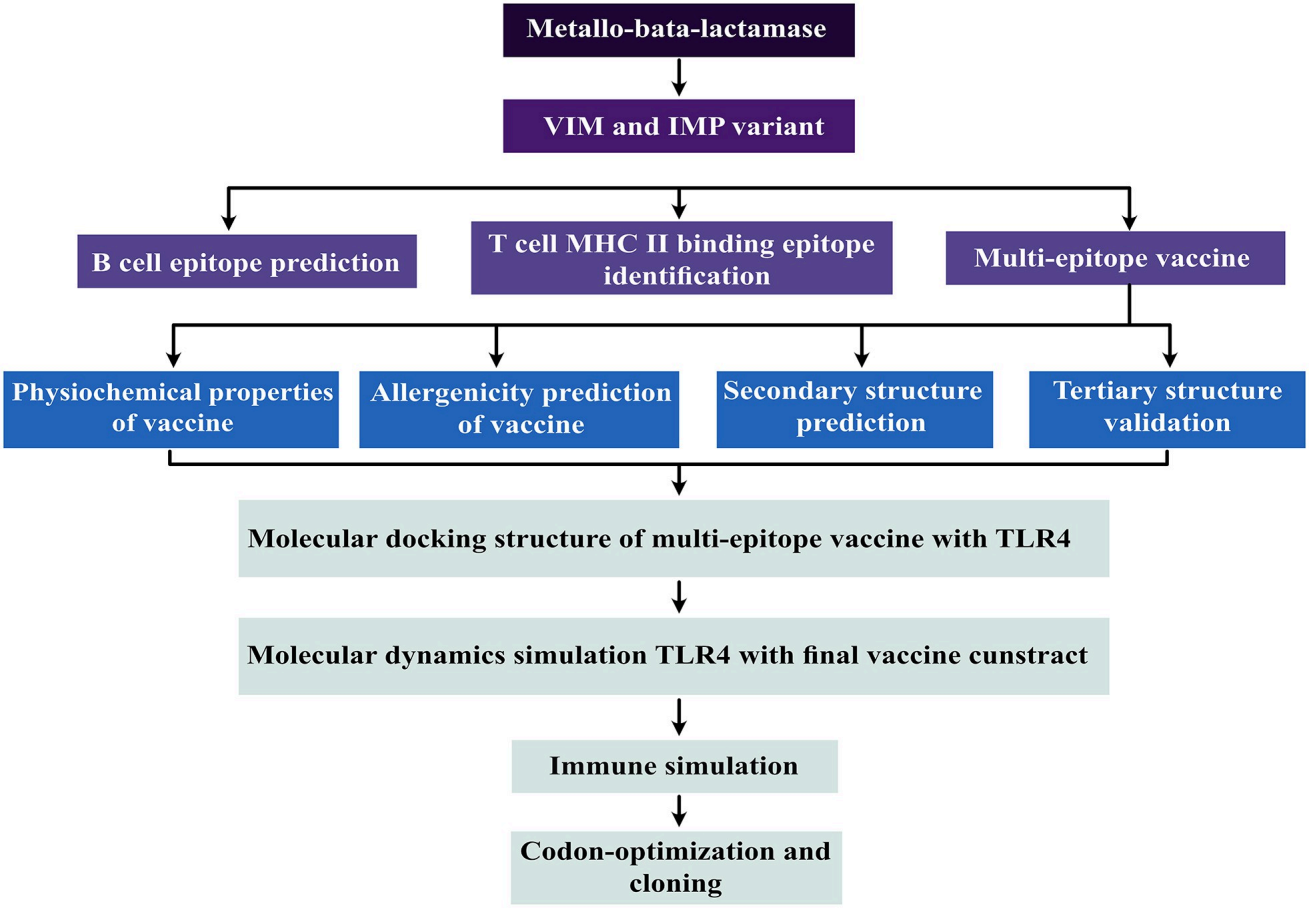
Schematic representation of the overall workflow applied in the current study.

The amino acid sequences and three dimensional (3D) structures of VIM and IMP variants were retrieved from the NCBI (https://pubmed.ncbi.nlm.nih.gov/) and Protein Data Bank (PDB) (https://www.rcsb.org/), respectively. Protein sequences were stored in FASTA format for further analysis. A flow chart representing the overall procedure from the antigen selection to vaccine construction and evaluation is illustrated in Fig 1.

### B-cell epitopes prediction

Linear B cell epitopes were assessed using the ABCpred and BCPred antibody epitope prediction tools. The ABCpred (http://www.imtech.res.in/raghava/abcpred/) server uses an artificial neural network to provide information about putative B-cell epitopes [34]. The antigenicity threshold parameter was considered as 0.51 (default), while the length of selected peptides for predicting the designed epitope was different. The BCPRED (http://ailab.ist.psu.edu/bcpred/predict.htm) server predicts and identifies the B cell epitope(s) using a novel method of a subsequence kernel [35]. In this study, peptide lengths of 12, 14, 16 and 18, which are present in both servers, were used to predict the desired epitopes. The reason for using different peptide lengths was to increase the probability of accessing to a high score immunogenic peptide with desirable specifications like non allergenicity and non-toxicity.

### T cell MHC class II binding epitopes identification

MHC class II binding predictions are widely used to identify epitope candidates in infectious agents, allergens, cancer, and autoantigens [36]. The Immune Epitope Database (IEDB) online server (http://tools.iedb.org/mhcii/) was applied for prediction of the MHC class-II binding sequences [36]. By default, we used the IEDB Recommended method to predict HLA-DR, HLA-DQ, and HLA-DP allele epitopes. We used the "HLA allele reference set" which contains 27 alleles that provide more than 99% of the population coverage for MHC II. In this study, MHC class II epitopes with a length of 15-mer were predicted. Other parameters such as IEDB Recommended, 15-mer length and adjusted rank sorting were set as default.

### Multi-epitope vaccine design

To construct the final vaccine, the epitopes predicted by different immunoinformatics software were linked together by separate linkers. HTL and B cell epitopes were joined together by GPGPG and KK linkers, respectively [37].

### Antigenicity, allergenicity and toxicity epitopes prediction

As a measure of the immune response, vaccine candidates were selected to predict antigenicity using the Vaxijen v2.0 server (https://www.ddg-pharmfac.net/vaxijen/) with a threshold value of 0.4 since the pathogen is a bacteria [38]. The server’s algorithm is mostly based on the method of sequence alignment and analyzes protein physiochemical properties to identify them as antigenic [39]. We used AllerTOP v.2.0 (https://www.ddg-pharmfac.net/AllerTOP/) servers [40] to predict the allergenicity of epitopes. Comparisons between different servers for allergen prediction shown that AllerTOP v.2 has significantly better than version 1 and has the highest accuracy with 88.7% [41]. ToxinPred server (http://crdd.osdd.net/raghava/toxinpred/) has been shown to be a unique method in silicon that will be useful in predicting the toxicity of peptides/proteins. This server performs well in designing toxic peptides and detecting toxic regions in proteins [42].

### Population coverage analysis of HLA MHC-II alleles

As it is known, the distribution of specific HLA alleles among different ethnicities and populations is an essential prerequisite for designing an epitope-based vaccine. To do this, the IEDB population coverage tool (http://tools.iedb.org/population/) was used to determine the population coverage of the best selected epitopes in several HLA alleles in different parts of the world [43].

### Physiochemical properties evaluation

ProtParam (toolhttps://web.expasy.org/protparam/protparam-doc.html) tool was used to analyze the physico-chemical properties of the protein sequence, including molecular weight, aliphatic score, in-vitro half-life instability index and grand average of hydropathy (GRAVY) [44]. GRAVY is calculated by dividing the total hydropathic values of all amino acids by the number of sequence residues. GRAVY indicates the amphipathic nature of proteins, negative and positive values indicate the hydrophilic and hydrophobic nature of the vaccine, respectively [45]. Protein solubility is an important feature in industrial and therapeutic applications. Protein-Sol (https://protein-sol.manchester.ac.uk/) is a web server for predicting protein solubility based on observing the two-dimensional distribution of protein solubility for *E*.*coli* proteins in cell-free expression [46]. According to this server, if our protein solubility score (QuerySol) was >0.45, it indicates that our protein is more soluble than the average soluble is *E*. *coli* protein.

### Secondary structure prediction

A web-based freely accessible online server called PSIPRED (http://bioinf.cs.ucl.ac.uk/psipred/) was used to determine the secondary structure of final vaccine construct [47]. To predict the secondary structures of proteins, this server is one of the most widely used servers, which uses two feed-forward neural networks to analyze the output obtained from PSI-BLAST [48].

### Refinement, validation and quality assessment of the tertiary structure

RaptorX server (http://raptorx.uchicago.edu/ContactMap/) was used to predict the modeling of the 3D structure based on the pattern [49]. The suggested time of this server to complete the processing of a sequence of 200 amino acids is defined as about 35 minutes. Meanwhile, based on the final vaccine construct, it predicts 5 models with different RMSDs. The server is designed to predict secondary protein structure, alignment quality assessment and sophisticated probable alignment sampling.

To further improve the quality of the third vaccine structure, the PDB file of the final vaccine generated from the RaptorX server was sent to the GalaxyRefine server (http://galaxy.seoklab.org/cgi-bin/submit.cgi?type=REFINE) for protein model structure refinement [50]. The server first reconstructs all side chain structures and repeatedly relaxes the structure with short molecular dynamics (MD) simulations after the side chain repackage perturbations. Parameters measured by GalaxyRefine server include global distance test-high accuracy (GDT-HA), root-mean-square deviation (RMSD), MolProbity (indicates crystallographic resolution), and Ramachandran favored score. The distance between the atoms is measured by RMSD. A lower RMSD value indicates better stability, and an RMSD score between 0 and 1.2 is usually acceptable [51].

UCLA-DOE LAB (https://saves.mbi.ucla.edu/) and ProSA-web (https://prosa.services.came.sbg.ac.at/prosa.php) servers were used to evaluate the validity and quality of the selected 3D structure [52, 53]. The UCLA-DOE LAB server has various tools such as PROCHECK and ERRAT for 3D structure validation. Ramachandran diagram was analyzed using PROCHECK section from UCLA-DOE LAB server (http://molprobity.manchester.ac.uk/). The Ramachandran diagram shows the statistical distribution of the combination of the backbone dihedral angles φ and ψ, as well as the percentage and number of residues in most favored, additional allowed, generously allowed, and disallowed region, which defines the quality of modeled structure [54]. ERRAT calculates an overall quality score for a specific input structure. If this score is outside the score range for native proteins, it means that the structure may contain some errors. A plot of local quality scores points to problematic parts of the model which are also highlighted in a 3D molecule viewer to facilitate their detection [52]. Then, a ProSA-web server was used that could detect errors in the final structure of the vaccine. This server shows the quality of the protein structure based on the measurement of total energy deviation [53].

### Molecular docking of multi-epitope vaccine with TLR4

Molecular docking is a computational method in structural molecular biology and computer-aided drug design. The purpose of ligand-protein binding is to predict the desired orientation of the ligand to the macromolecular target (receptor) to form a stable complex [55, 56]. The designed vaccine was docked with the TLR4 using PatchDock server (https://bioinfo3d.cs.tau.ac.il/PatchDock/) to evaluate the capacity of the vaccine to induce innate immunity [57]. This server is a geometry-based molecular docking algorithm, where both receptor and ligand molecules are given either by PDB molecule or by loading a file in PDB format [57]. Consequently, the top 10 results of the PatchDock server were evaluated using the FireDock (https://bioinfo3d.cs.tau.ac.il/FireDock/) server [58] to calculate the Global binding energy that consists of attractive and repulsive van der Waals (VdW) forces, atomic contact energy (ACE) and hydrogen bond [59]. In this study, different TLRs including TLR1, TLR1-TLR2 heterodimer, TLR2, TLR4, and TLR4/MD-2 heterodimer were used, which are a total of six protein database (PDB) identifiers from the RCSB PDB database, including 6NIH, 2Z7X, 2Z80, 2Z62, 2Z63, and 3FXI were selected. Receptors and vaccine structure were prepared by removing heteroatoms such as H2O molecules, ligands, then polar hydrogens and Kollman charges were added to them. In addition, visualization of the vaccine-TLR4 complex interactions was performed by LigPlot+ software.

### Molecular dynamic simulation

Molecular dynamics (MD) simulation was performed to investigate the stability of the vaccine-TLR4 complex using GROMACS v5.0 software [60]. GROMACS is an open source software widely used for dynamic simulation of biomolecules. This software provides a rich set of calculations, preparation and analysis tools. Various parameters such as RMSD, RMSF (root mean square fluctuations), Rg (Radius of gyration), and SASA (Solvent Accessible Surface Area) were performed for MD simulation. Solvation was carried out by SPCE water model followed by electro-neutralization and energy minimization under steepest descend algorithm. Temperature and pressure were both coupled in 310 k and 1 bar respectively. Temperature and pressure were both coupled in 310 k and 1 bar respectively. A nose-hoover thermostat and a Parinello- Rahman barostat were used in NVT and NPT ensembles respectively. Bond constraints were done by linear constraints Solver (LINCS) method and van der Waals and electrostatic non-bonded interactions were measured in a cutoff of 1 nm. Finally, a 100 ns MD simulation of selected vaccine-TLR4 complex was considered under the leap-frog algorithm.

### Normal mode analysis (NMA)

Normalized mode analysis (NMA) is a technique that can be used to describe the flexible states accessible to a protein about its equilibrium position [61]. MD simulation and NMA are two useful methods to describe different dynamic aspects of biological macromolecules. Meanwhile, MD method is more accurate than NMA. Although NMA is based on normal mode vibrations, which are defined as simple harmonic oscillations around an energy minimum, MD can encompass a significantly larger volume of conformational space. However, many studies have shown that the results obtained from NMA can describe dynamic aspects of biomolecules well [62]. In the present study, the vaccine-receptor complex was uploaded in PDB file format to the iMODS server (http://imods.chaconlab.org/) [63]. iMODS server is based on NMA in internal (dihedral) coordinates, which can predict collective motions of macromolecules, including proteins. This server presents the results in the form of graphs by calculation of deformability, B-factor, eigenvalues, variance, covariance map and elastic network.

### Immune simulation

C-IMMSIM server (http://kraken.iac.rm.cnr.it/C-IMMSIM/index.php?page=1) is used to Immune simulation of a generic protein sequence in the form of its amino acid sequence. This online server defines a mammalian immune system’s both humoral and cellular response to the vaccine construct [64]. In summary, C-IMMSIM displays images in which the major classes of cells of both the lymphoid (T helper lymphocytes (Th), cytotoxic T lymphocytes (CTL), B lymphocytes, and antibody-producer plasma cells, PLB) and the myeloid lineage (macrophages (M) and dendritic cells) are represented [64]. C-IMMSIM has parameters such as injection schedule in terms of time and dose, host haplotype selection, simulation volume and random seed that determine the characteristics of the initial population of lymphocytes. The simulated parameters in this study included: a) a vaccine without LPS, b) considering three doses of vaccine (to create an efficient and long-lasting immune response) with time intervals of 1, 84 and 168 c) the volume of the simulation and the simulation steps were adjusted to 10 and 1100, respectively. Other perimeters remain unchanged.

### Codon-optimization and cloning for design multi-epitope vaccine

Java Codon Adaptation Tool (JCat) server (http://www.prodoric.de/JCat) was used to quantify the expression level of the multi-epitope vaccine in *E*. *coli* (strain K12). Jcat calculated the GC content and Codon Adaptation Index (CAI) value for the query sequence in order to ensure maximum expression [65]. The final vaccine was then cloned into pET-28a (+) plasmid using SnapGene software (version 5.2.3) (https://www.snapgene.com/).

### Analysis of the vaccine mRNA

RNAfold (http://rna.tbi.univie.ac.at/cgi-bin/RNAWebSuite/RNAfold.cgi) web server was used to predict the secondary structure of mRNA. This server predicts the minimum free energy (MFE) produced thermodynamically of the query mRNA structures [66]. After obtaining the optimized DNA sequence through the JCat server, for analysis of mRNA folding and vaccine secondary structure, first converted into a potential DNA sequence by DNA<->RNA->Protein at http://biomodel.uah.es/en/lab/cybertory/analysis/trans.htm.

## Results

### Primary analysis of the candidate sequences

In the present study, the amino acid sequences of VIM and IMP variants were retrieved from the NCBI server and shown in Table 1. All complete (not partial) sequences retrieved using the Clustal Omega server (https://www.ebi.ac.uk/Tools/msa/clustalo/) were aligned with the default parameters and prepared for further analysis.

**Table 1:** NCBI accession numbers of VIM and IMP proteins in bacterial species.

Accession numbers (VIM)	Organism	Accession numbers (IMP)	Organism
AVI26303.1	*Pseudomonas aeruginosa*	WP_003159548.1	*Proteobacteria*
ABC97285.1	*Pseudomonas aeruginosa*	WP_015060105.1	*Gammaproteobacteria*
ACL50513.1	*Pseudomonas aeruginosa*	WP_032492096.1	*Gammaproteobacteria*
ACY29468.1	*Escherichia coli*	WP_032490175.1	*Pseudomonas*
CBY80143.1	*Klebsiella pneumoniae*	WP_063860584.1	*Pseudomonas aeruginosa*
AEI25539.1	*Pseudomonas aeruginosa*	WP_060614779.1	*Gammaproteobacteria*
RTQ94426.1	*Klebsiella pneumoniae*	WP_063860594.1	*Enterobacteriaceae*
AEZ49857.1	*Klebsiella oxytoca*	WP_063860598.1	*Pseudomonas aeruginosa*
AGS82586.1	*Klebsiella pneumoniae*	WP_063860613.1	*Escherichia coli*
WP_063865192	*Klebsiella pneumoniae*	WP_094009805.1	*Escherichia coli*
WP_063865193.1	*Pseudomonas aeruginosa*	WP_085562391.1	*Escherichia coli*
WP_094009807.1	*Klebsiella pneumoniae*	WP_114699280.1	*Pseudomonas aeruginosa*
APY16324.1	*Serratia marcescens*	WP_114699281.1	*Pseudomonas aeruginosa*
ATY69436.1	*Klebsiella pneumoniae*	WP_114699282.1	*Pseudomonas aeruginosa*
BBE07875.1	*Pseudomonas aeruginosa*	WP_116786839.1	*Pseudomonas aeruginosa*
AWY71041.1	*Citrobacter freundii*	WP_122630861.1	*Pseudomonas aeruginosa*
WP_140423327.1	*Klebsiella pneumoniae*		
QHI08146.1	*Pseudomonas aeruginosa*		
QYZ89894.1	*Proteus mirabilis*		
WP_142836723.1	*Proteus mirabilis*		
UBL87558.1	*Klebsiella oxytoca*		

### B-cell epitopes prediction

Linear B-cell epitopes were predicted using ABCpred and BCPRED server, respectively. Scores above 0.80 were considered for selecting epitopes on these two servers. Finally, 3 epitopes (HDDRVGGVDVLR, EAEVVIPGHGLPGG, AVRFGPVELFYP) from VIM and 4 epitopes from IMP (VFYPGPGHTPDN, KIEVFYPGPGHTPD, DLKIEKLDEGVYVHTS, IPTYASELTNELLKKDGK) with different lengths of 12, 14, 16 and 18 were selected (Table 2).

**Table 2:** Most conserved B cell epitope sequences predicted by these two methods (BCPREDS and ABCpred).

Protein	Server	length	Start	End	Epitope	Score	Antigenicity (Threshold: 0.4)	Allergenicity	Toxicity
**VIM**	BCPred	12	111	122	HDDRVGGVDVLR	0.845	1.2038	NON-ALLERGEN	Non-Toxin
		14	222	235	EAEVVIPGHGLPGG	0.997	0.4365	NON-ALLERGEN	Non-Toxin
	ABCpred	12	157	168	AVRFGPVELFYP	0.81	0.89	NON-ALLERGEN	Non-Toxin
**IMP**	BCPred	12	139	150	VFYPGPGHTPDN	1	0.8482	NON-ALLERGEN	Non-Toxin
		14	136	149	KIEVFYPGPGHTPD	0.985	1.0192	NON-ALLERGEN	Non-Toxin
	ABCpred	16	23	38	DLKIEKLDEGVYVHTS	0.83	0.9629	NON-ALLERGEN	Non-Toxin
		18	103	120	IPTYASELTNELLKKDGK	0.82	0.5238	NON-ALLERGEN	Non-Toxin

### MHC class II binding epitopes prediction

We selected a total of 4 MHC class II epitopes from both VIM and IMP proteins, with a length of 15 amino acids (Table 3). Among these epitopes, 2 epitopes VIM (VGGVDVLRAAGVATY and TSAGNVADADLAEWP) and 2 epitopes from IMP (FFIFLFCSIATAAEL and ERGYKIKGSISSHFH) met the criteria for selection of antigen, non-allergenicity and non-toxicity.

**Table 3:** Most probable predicted epitopes with MHC class II alleles from IEDB analysis tool.

Protein	Peptide sequence	Start	End	Allele	Antigenicity (Threshold: 0.4)	Allergenicity	Toxicity	Conservancy (%)
**IMP**	FFIFLFCSIATAAEL	8	22	HLA-DRB1*01:01	1.814	NON-ALLERGEN	Non-Toxin	100%
				HLA-DRB1*04:01				
				HLA-DRB1*04:05				
				HLA-DRB1*08:02				
				HLA-DRB1*15:01				
				HLA-DPA1*01:03/DPB1*02:01				
				HLA-DQA1*04:01/DQB1*04:02				
				HLA-DPA1*02:01/DPB1*01:01				
				HLA-DPA1*03:01/DPB1*04:02				
				HLA-DQA1*03:01/DQB1*03:02				
				HLA-DPA1*02:01/DPB1*14:01				
	ERGYKIKGSISSHFH	76	90	HLA-DRB1*04:01	0.9222	NON-ALLERGEN	Non-Toxin	100%
				HLA-DRB1*08:02				
				HLA-DRB1*07:01				
				HLA-DRB1*09:01				
				HLA-DRB1*11:01				
				HLA-DRB1*13:02				
				HLA-DRB3*02:02				
				HLA-DPA1*02:01/DPB1*14:01				
**VIM**	VGGVDVLRAAGVATY	115	129	HLA-DRB1*01:01	0.5207	NON-ALLERGEN	Non-Toxin	100%
				HLA-DRB1*09:01				
				HLA- DRB1*12:01				
				HLA-DRB1*13:02				
				HLA-DQA1*01:02/DQB1*06:02				
				HLA-DQA1*05:01/DQB1*03:01				
	TSAGNVADADLAEWP	197	211	HLA-DQA1*03:01/DQB1*03:02, HLA-DQA1*04:01/DQB1*04:02, HLA-DQA1*05:01/DQB1*02:01	0.8369	NON-ALLERGEN	Non-Toxin	100%

### Physicochemical and other evaluations of the multi-epitope vaccine

**Fig 2:**
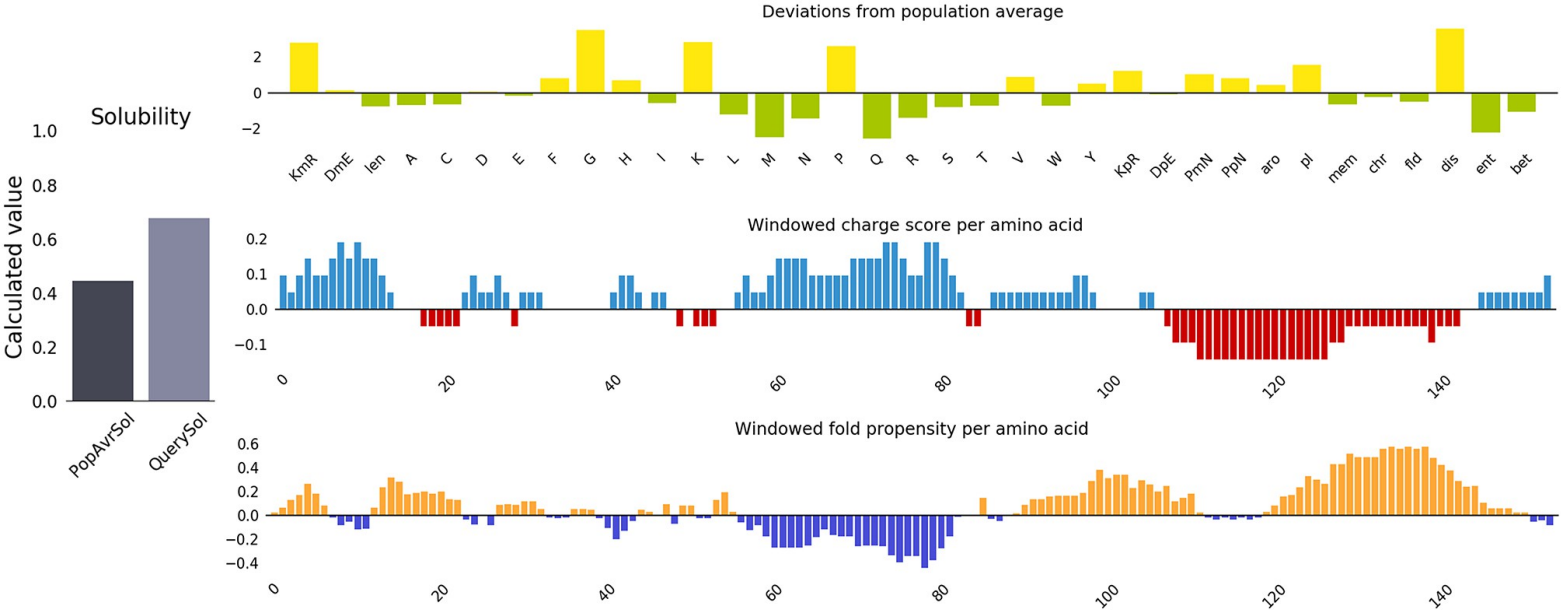
The solubility analysis of the vaccine construct by Protein-Sol server was predicted to be 0.679.

The molecular weight of the construct was 18476.15 Daltons and estimated half-life of 3.5 hours (mammalian reticulocytes, in vitro), 10 min (yeast, in vivo), and more than 10 hours (*Escherichia coli*, in vivo). The instability index was calculated 13.82 (<40) for vaccine stability. The aliphatic index was calculated 77.30, so the vaccine is considered as a thermostable protein. The theoretical pI of the vaccine was calculated to be 9.03. The GRAVY index of the vaccine was -0.370, which reflects the vaccine’s polar nature and effective interaction with water, suggesting high solubility. In addition, the number of residues with negative charge (Asp + Glu) and positive charge (Arg + Lys) were determined to be 21 and 25, respectively. Finally, C843H1317N225O240S1 was obtained as the vaccine formula. According to Fig 2, the solubility of the vaccine construct in terms of QuerySol (scaled solubility value) was 0.679.

#### Population coverage of selected epitopes

Evaluating the frequency of distribution of HLA alleles in 15 different geographical regions of the world were examined [43]. The highest coverage was observed in East Asia (79.75%), North America (78.55%) and Europe (76.02%), followed by South Asia (66.16%), West Indies (64.09%), North Africa (61.95%), East Africa (59.93%), West Africa (59.33%), Oceania (59.9%), Northeast Asia (55.84%), Central Africa (54.2%), South America (52.63%), Southeast Asia (51.48%) and Central America (47.27%). And the lowest coverage was observed in South Africa (7.65%).

### Multi-epitope vaccine design

Epitopes that were highly antigenic, non-allergenic, and non-toxic were considered suitable vaccine candidates and were eventually selected for constructing the vaccine. The final epitopes were connected using KK and GPGPG flexible linkers. The sequence of the multi-epitope vaccine was resulted as: “HDDRVGGVDVLRKKAVRFGPVELFYPKKEAEVVIPGHGLPGGKKDLKIEKLDEGVYVHTSKKIPTYASELTNELLKKDGKKKKIEVFYPGPGHTPDNKKVGGVDVLRAAGVATYGPGPGTSAGNVADADLAEWPGPGPGFFIFLFCSIATAAELGPGPGERGYKIKGSISSHFH”.

### Antigenicity, allergenicity and toxicity evaluation

Antigenicity of the final vaccine construct was predicted 0.9098% by VaxiJen at 0.4% threshold for bacterial model. Allergenicity and toxicity were evaluated based on AllerTOP 2.0 and ToxinPred, respectively. According to the results obtained from AllerTOP 2.0 and ToxinPred, the vaccine is likely to be allergenic and non-toxic (Table 3).

### Secondary structure prediction

**Fig 3:**
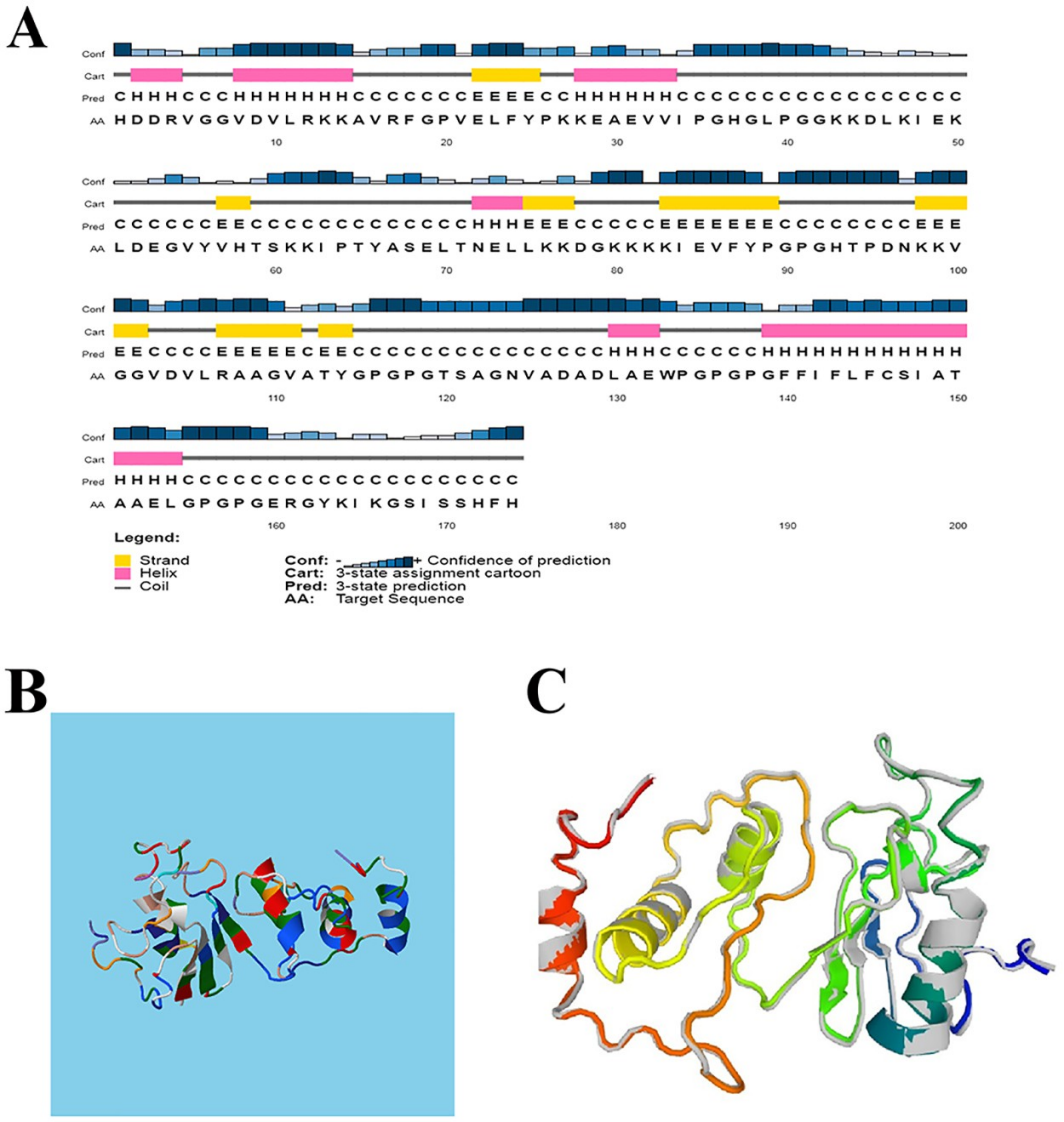
Displays the second and 3D of the designed multi-epitope vaccine. (**A**) The alpha helix residues are in pink, the beta- strand residues are in yellow and the coil residues are in grey. The predicted secondary structure indicates that the final vaccine constitutes 21.84% alpha-helix, 16.09% beta-strand, and 62.07% coil, respectively. (**B**) The 3D structure of the designed vaccine construct. **(C)** Model of the 3D structure of the refined vaccine by the GalaxyRefine server.

The predicted structure of the PSIPRED server represents 21.84% alpha-helix, 16.09% beta- strand and 62.07% coil structure as shown in Fig 3A.

### Refinement, validation and quality assessment of the tertiary structure

From the five models proposed by the RaptorX server, the structure of Model 1 (Fig 3B) with an estimated RMSD of 5.7610 Angstrom (Å) was selected. According to the GalaxyRefine server results, the best refined model (2) is shown in Fig 3C with a GDT-HA score of 0.9569, a RMSD score of 0.399, a MolProbity score of 1.913, a Clash score of 8.7 and a Ramachandran score of 93 (Table 4). Therefore, it can be concluded that the quality of the refined model is high compared to the raw structure.

**Table 4:** Quality scores of 5 models predicted by GalaxyRefine server.

Model	GDT-HA	RMSD	MolProbity	Clash score	Rama favored (%)
**Initial model**	1	0.000	2.239	14.3	89
**Model 1**	0.9526	0.417	1.953	9.1	92.4
**Model 2**	0.9569	0.399	1.913	8.7	93
**Model 3**	0.9612	0.382	2.066	10.9	91.3
**Model 4**	0.954	0.408	1.885	6.8	91.3
**Model 5**	0.9483	0.424	2.054	7.5	91.3

**Fig 4:**
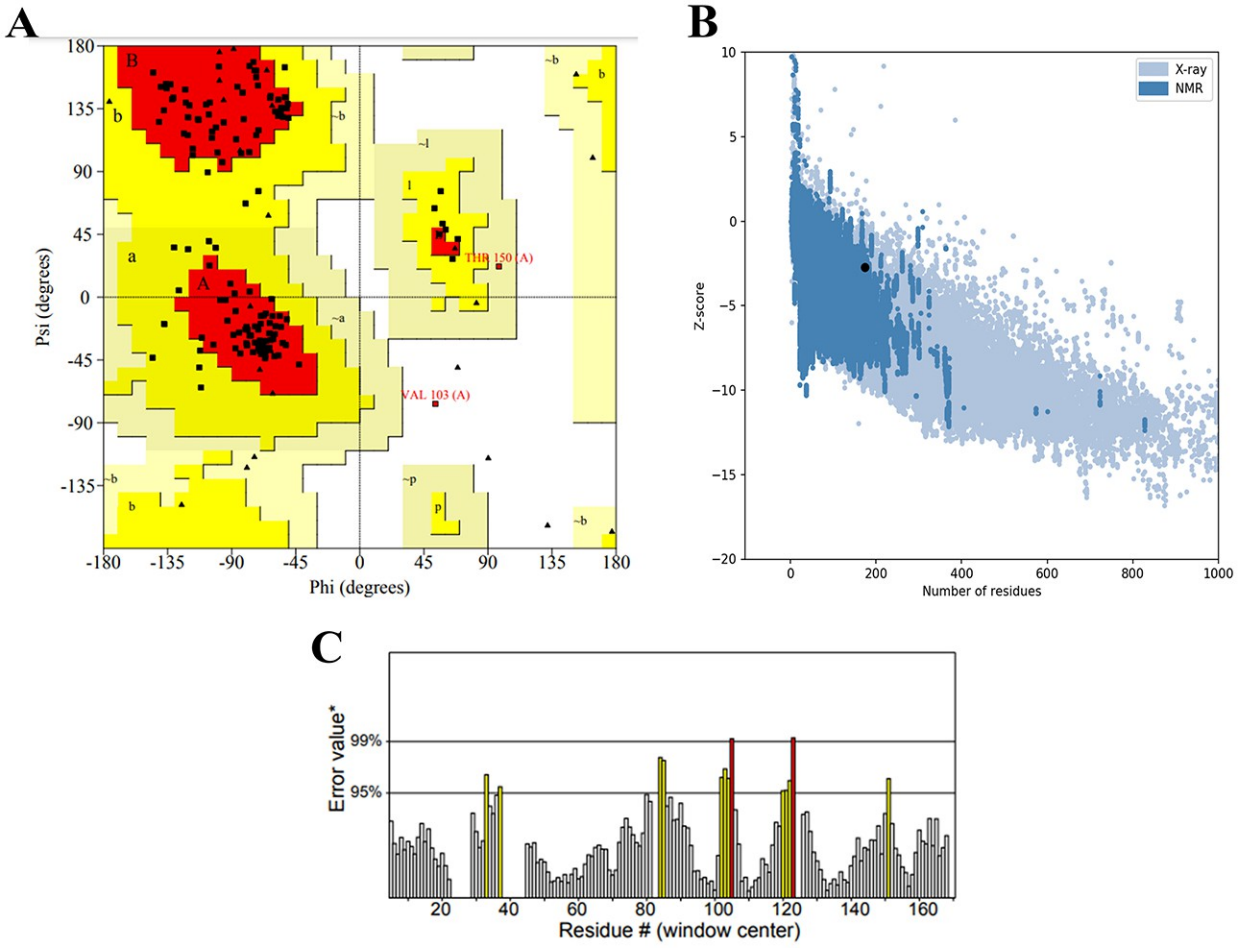
**(A)** Ramachandran plot analysis based on final vaccine epitopes showing 83.2%, 15.3%, 0.8% and 0.8% of protein residues in favored, additional allowed, generously and disallowed (outlier) area region respectively. **(B)** ProSA-web results indicated a Z-score of– 2.71. The z-score of protein vaccine is showed in large black spot. Z-score plot consists the Z-scores of all experimentally protein chains in PDB determined by nuclear magnetic resonance (NMR) spectroscopy (dark blue) and X-ray crystallography (light blue). **(C)** Validation of the vaccine structure by ERRAT with a score of 91.156.

Ramachandran plot analysis based on final vaccine epitopes showing 83.2%, 15.3%, 0.8% and 0.8% of protein residues in favored, additional allowed, generously and disallowed (outlier) area region respectively (Fig 4A). While ProSA-web has shown the Z-score of the vaccine candidate as -2.71 (Fig 4B). ERRAT server was used to analyze the statistics of non-bonded interactions. The ERRAT score was set at 91,156 (Fig 4C). Generally, an ERRAT score greater than 50 represents a good quality model [67] and so, the score 91.156 validates our modeled structure.

### Molecular docking of multi-epitope vaccine with TLR4 receptor

**Fig 5:**
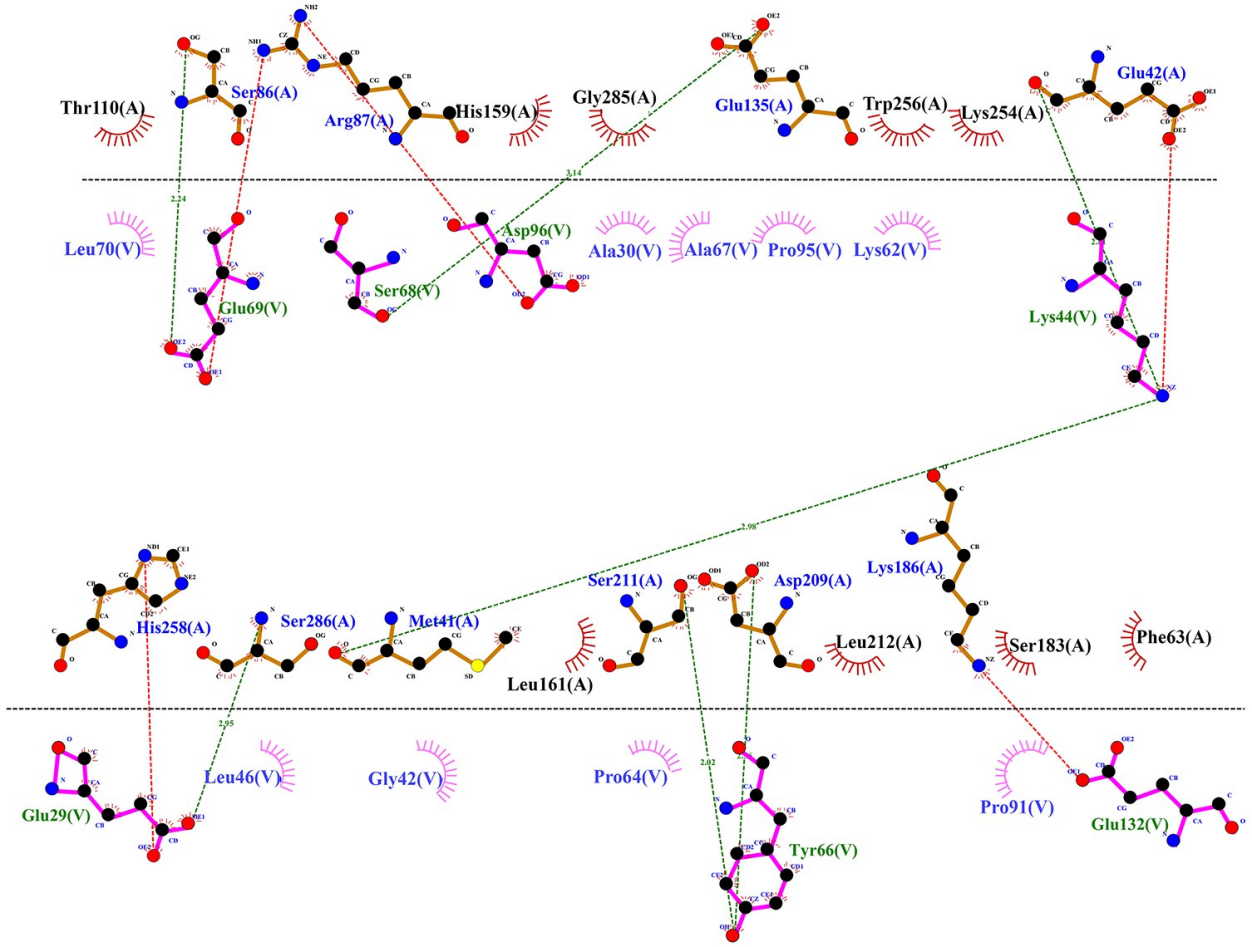
Hydrophobic and hydrogen bond forming residues in the best docked conformations. Hydrogen bonds and salt bridges are shown with green and red dashed lines, respectively. Half circle in red color indicates the residues involved in hydrophobic interactions.

Among the proposed TLRs, the results of molecular docking of vaccine construct with TLR4 were selected using a PatchDock server. Afterwards, the Global Energy of the top ten results were determined by FireDock (Table 5 and S1 File). Vaccine construct and TLR4 interaction score was obtained 13176 and Global Energy was -25.35 kilocalories per mole (kcal/mol). The schematic diagram of the interaction between vaccine construct and TLR4 was generated by LigPlot+software (Fig 5). Hydrogen bonds, salt bridges, and hydrophobic interactions were obtained by the DIMPLOT program. DIMPLOT was shown, Ser68, Lys44, Glu29, Tyr66, and Glu132 residues from TLR4 were potentially bounded to, Glu135, Glu42, Ser286, Asp209, and Ser211 residues from designed construct through hydrogen bonds, in addition, Gln69, Asp96, Glu29 and Glu132 residue from TLR4 were bounded to Ser86, Arg87, His258 and Lys186 from construct through salt bridges interaction.

**Table 5:** Global energy designated from the top ten results by the FireDock server.

Solution No	Score	Global Energy	Attractive VdW	Repulsive VdW	ACE	HB
**1**	14798	-18.76	-16.19	10.34	0.97	-0.52
**2**	14610	-15.28	-23.6	19.9	8	-5.62
**3**	14190	-22.94	-37.6	30.72	0.04	-4.25
**4**	14046	-9.62	-22.36	8.23	3.79	-2.81
**5**	13496	-19.34	-26.41	18.82	12.36	-3
**6**	13360	5.99	-25.99	13.28	9.69	-3.57
**7**	13188	527.35	-33.29	665.22	10.23	-3.55
**8**	13176	-25.35	-34.43	12.04	13.44	-5.73
**9**	13102	28.96	-16.07	11.5	6.65	-0.91
**10**	13098	26.29	-36.11	46.95	16.55	-2.32

### Vaccine-TLR4 complex MD simulations

The vaccine-TLR4 complex was dynamically simulated for a 100 ns of MD simulation in order to confirm stability of complex in dynamic state. Various analysis such as RMSD, RMSF, Rg, and SASA were carried out for MD simulation.

**Fig 6:**
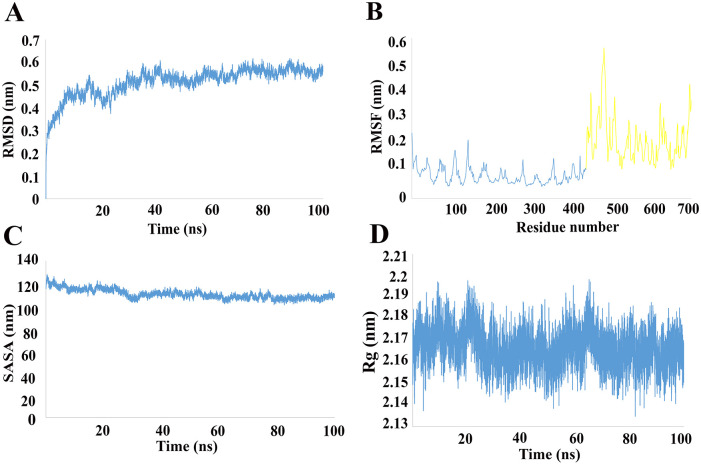
Comparison of changes in RMSD, RMSF, Rg, SASA values of protein in interaction with TLR4.

RMSD is widely used in the analysis of macromolecular structures and dynamics. Two basic parameters can be understood based on the pattern of the RMSD plot: a) whether the system has reached the equilibrium state or not, and b) whether the simulation time was sufficient or not. The RMSD diagram of simulated complex is reached to a platue condition which confirms the equilibration of the system and also indicate that the simulation time is sufficient for this protein in these situation (Fig 6A). Furthermore, lack of extreme fluctuations in the RMSD plot indicates the stability of the ligand-receptor complex in dynamic state. The fluctuation of each protein residue was analyzed using RMSF. The overall residue fluctuation of ligand is higher than the receptor, which the probable reason is the higher ratios of turns and coils in its structure (Fig 6B).

**Fig 7:**
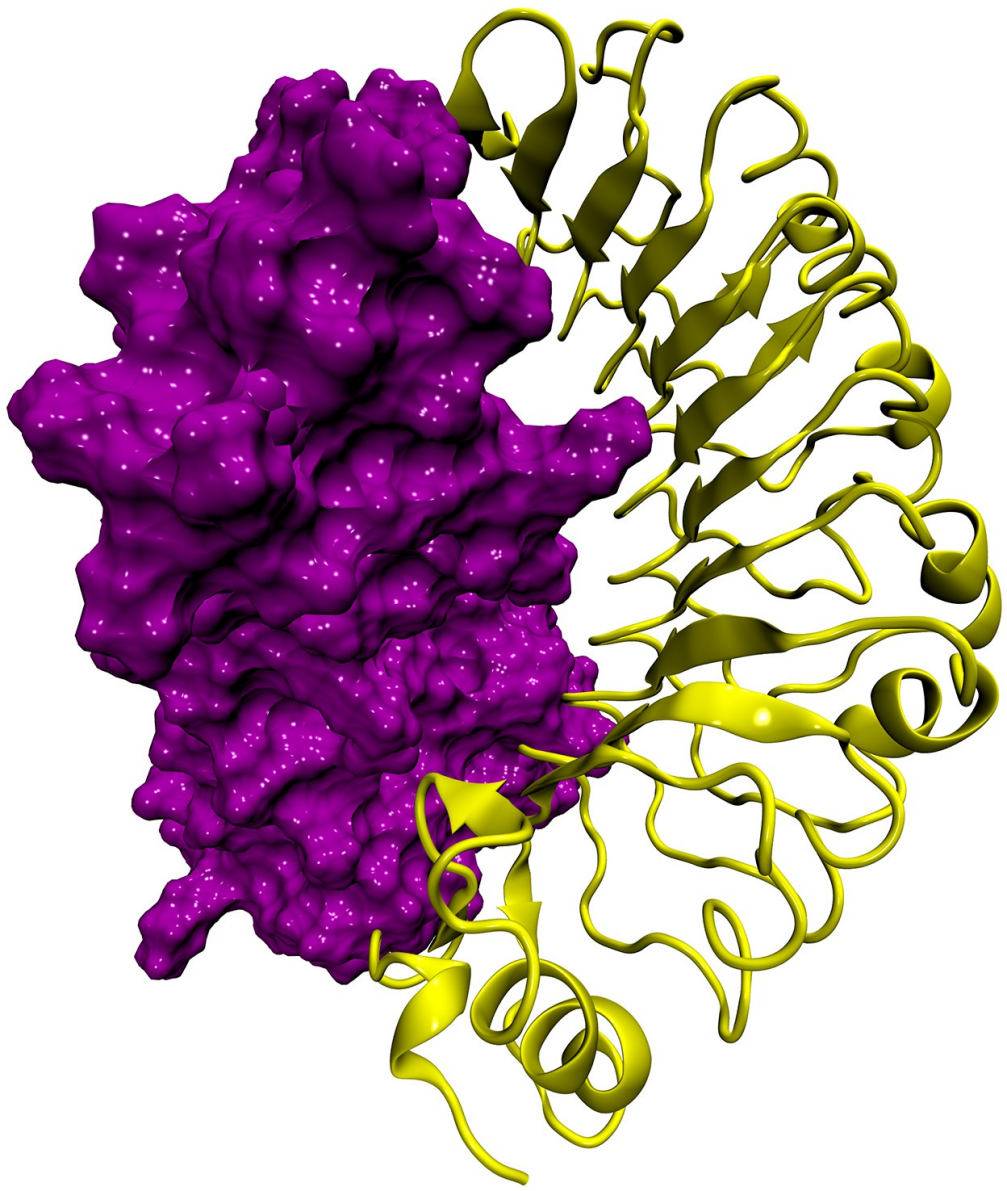
MD simulation vaccine construct (purple) complexed with TLR4 immune receptor (yellow).

SASA of the simulated complex decreased during the simulation time (Fig 6C). Also, the radius of gyration for the ligand-receptor complex was around 2.19 nm (Fig 6D). These results indicate that the receptor-ligand complex is compact and the components getting closer and form more stable interactions. The final conformation of vaccine construct and TLR4 complex is shown in Fig 7. Altogether, it is shown that the final construct-TLR4 complex has a good stability in solvated dynamic state.

### NMA evaluation of the vaccine-receptor complex

**Fig 8:**
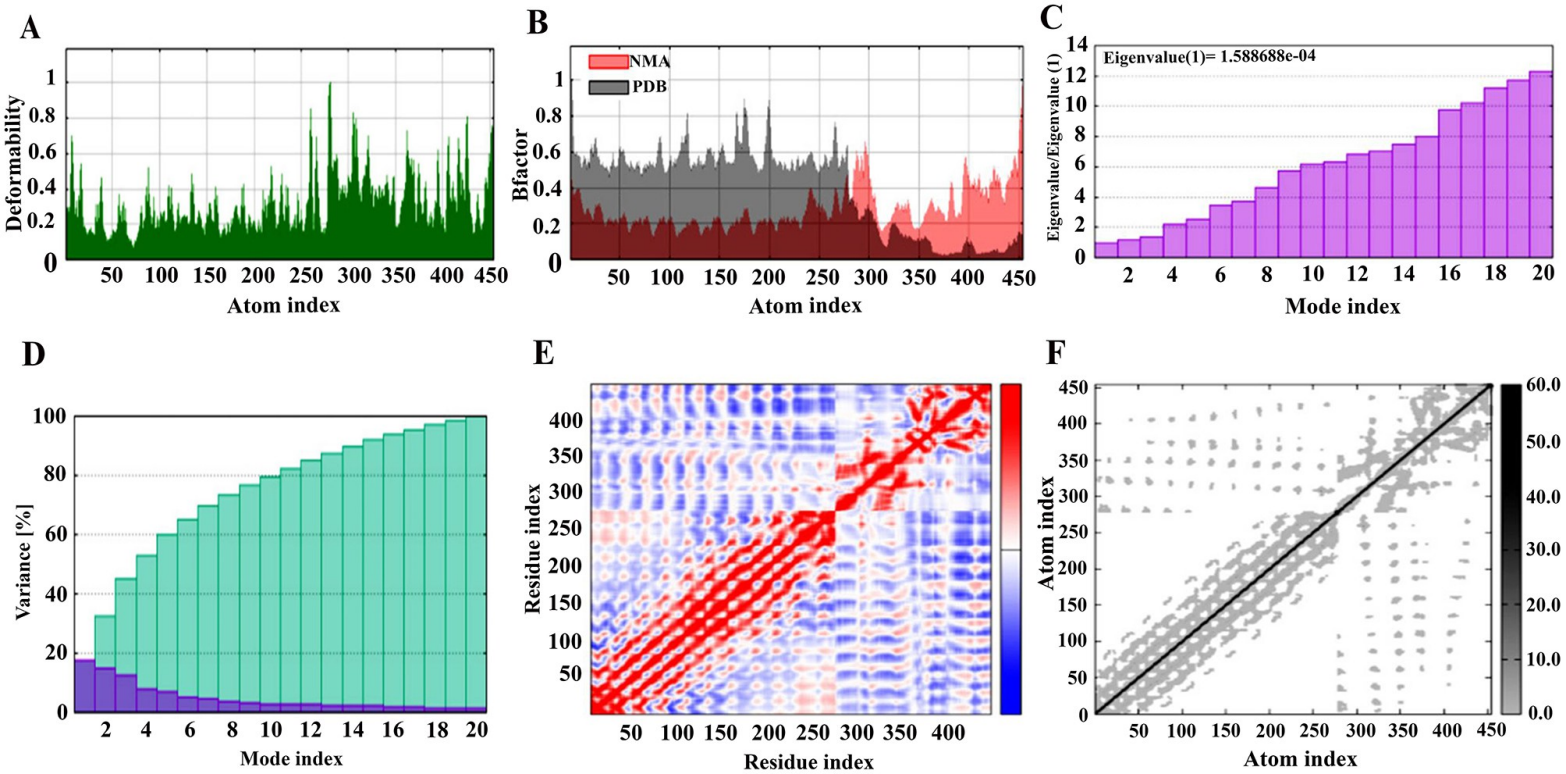
The molecular dynamics simulation of the vaccine-TLR4 docked complex. (A) Main-chain deformability simulation, the hinges are regions with high deformability. (B) B-factor values calculated by normal mode analysis, quantifying the uncertainty of each atom. (C) The eigenvalue of the docked complex, showing the energy required to deform the structure. (D) The covariance matrix between pairs of residues (red: correlated, white: uncorrelated, blue: anti-correlated). (E) The elastic network model, suggesting the connection between atoms and springs. The springs are more rigid if their greys are darker.

The NMA of the vaccine-receptor complex is shown in Fig 8. Fig 8A shows the deformation of the protein flexibility, which depends on the individual distortion of each residue depicted by the chain hinges. On the other hand, locations with hinges are areas with high deformability and illustrates a stable binding. B-factor (Fig 8B) shows the relative amplitude of atomic displacement with respect to the equilibrium position. According to Fig 8B, few fluctuations of atomic displacement were observed for the vaccine-TLR4 complex. The eigenvalue graph is depicted in (Fig 8C) and its numerical value is estimated 1.588688e-04. Also the variance graph corresponding to normal mode is presented in (Fig 8D). Fig 8E shows a covariance matrix map of the interaction between residue pairs of the proteins of a complex (red: correlated motion between a pair of residues, white: non-correlated motion, and blue: anti-correlated motion). Finally, the stiffness study of the protein complex was performed using elastic network analysis. As shown in Fig 8F, the darker the gray dots, the greater the protein stiffness in certain sections.

### Immune simulation

**Fig 9:**
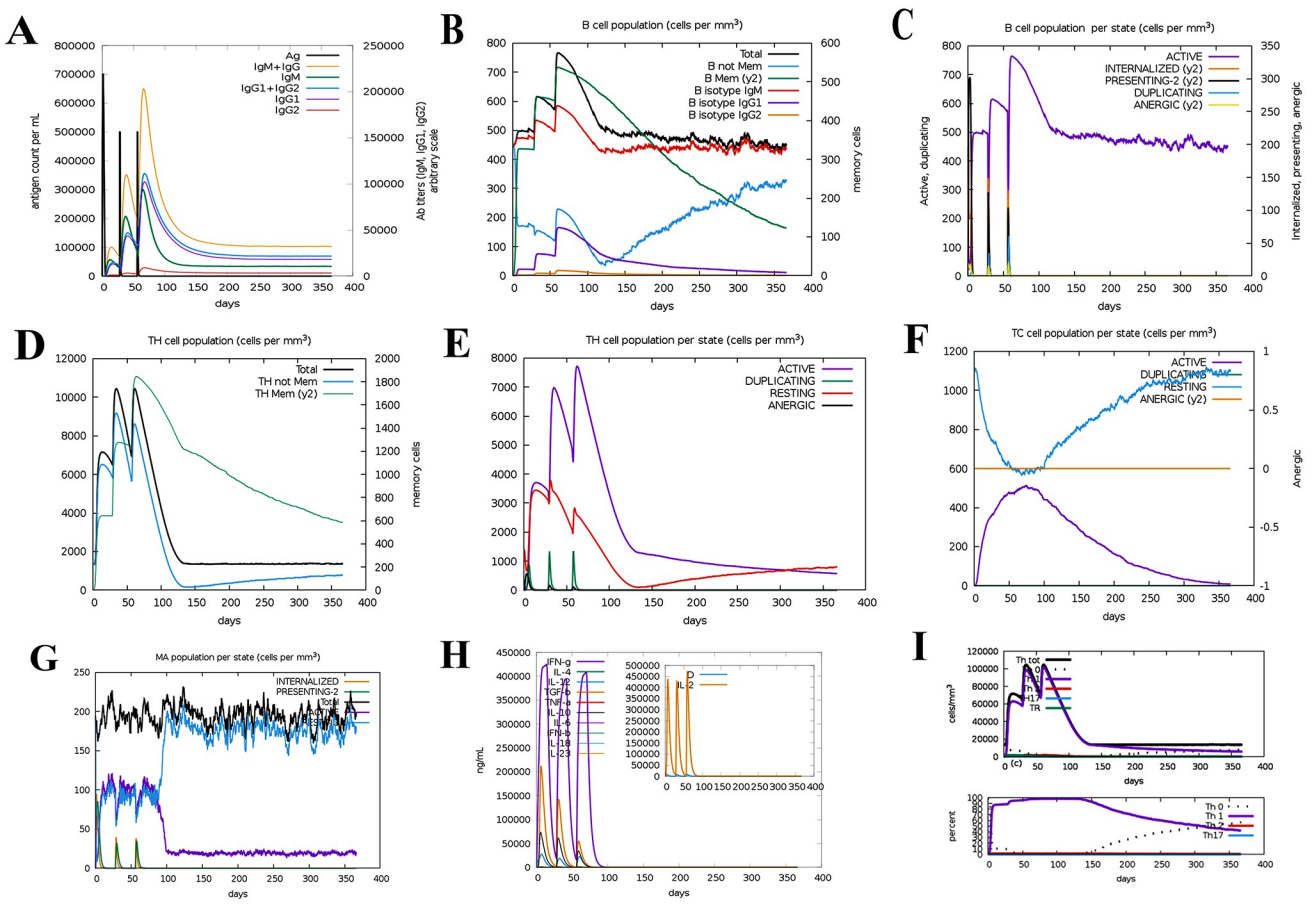
Immune simulation results by C-ImmSim. **(A)** Immunoglobulin/antibodies production in response to antigen injection. Various subtypes of immunoglobulin are represented as colored peaks. **(B)** Display B cell population after three injections. **(C)** population per state of B-cell. (D) T-helper cell population (indicates a substantial increase in TH memory cell). **(E)** population per state of T-helper cell. **(F)** Production of cytotoxic-T cells after vaccine injection. **(G)** population per state of macrophages. **(H)** Production of cytokines and interleukins (increased production of IFN-γ and IL-2) after vaccination. **(I)** Significant increase in Th1.

Based on Fig 9(A), high immunoglobulin titers were produced after the first and second injections of the vaccine. The increase in IgM concentration in the initial response was found to be normal because IgM is the first type of antibody produced. During the simulation period, antibody levels (IgM+IgG, IgG1+IgG2, IgM, IgG1) increased in the secondary and tertiary responses, leading to a decrease in antigen levels (Fig 9A). In Fig 9(B), after vaccination, we saw a sharp increase in the population of B cells, including memory B cells, which indicates the potential for isotype switching and memory formation. Fig 9(C) shows the increase in cell proliferation in B cells as well as the presentation of antigen following vaccine injection. According to (Fig 9D–9F), the levels of TH (helper) and TC (cytotoxic) cell populations also increased significantly with respect to memory development. As shown in Fig 9(G), we see an increase in macrophage activity and antigen presentation during the vaccine injection phase. A significant increase in interferon gamma (IFN-γ) titer (Fig 9(H)) and a moderate increase in interleukin-2 (IL-2) (Fig 9(H)) are observed after the third injection of the vaccine. Finally, in Fig 9(I), we saw a significant increase in Th1. These data emphasize the possibility of our candidate vaccine being able to induce an effective immune response capable of protecting against disease.

### Codon-optimization and in silico cloning

**Fig 10:**
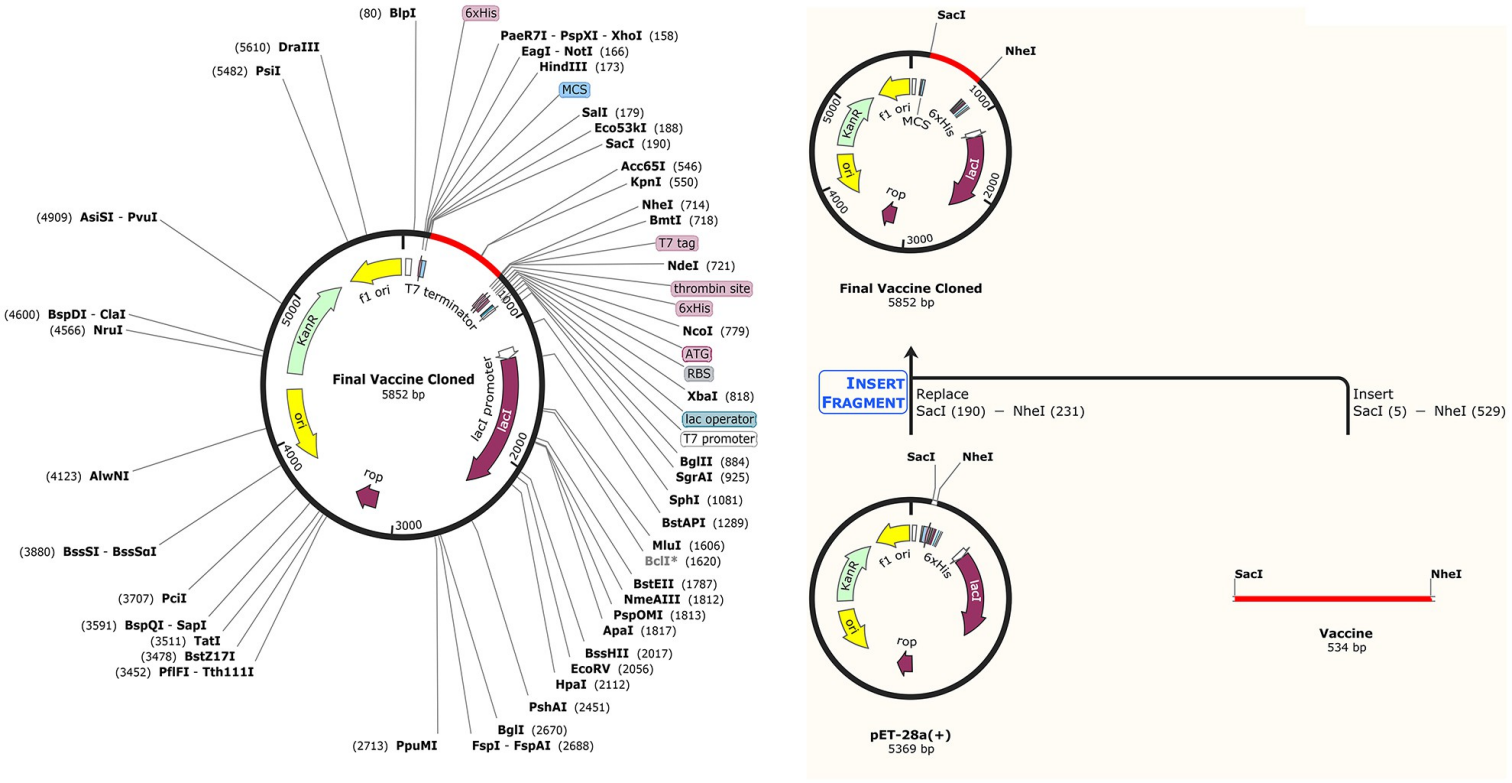
*In silico* cloning of the final vaccine construct into pET28a (+) between the SacI and NheI restriction enzyme sites. Here, the red areas indicate the candidate vaccine against VIM and IMP, and the black areas represent the expression vector, pET28a (+).

The final multi-epitope vaccine was codon-optimized for *E*. *coli* strain K12 using the JCAT server. The optimized codon sequence had a length of 524 nucleotides. The results showed that our vaccine had a CAI of 1.0 and a GC content of 52%, which is very close to the GC-Content of *E*. *coli* K12 strain with 50.73. SacI (GAGCTC) and NheI (GCTAGC) restriction sites were added to the N and C terminals of the final vaccine codon sequence. Then, SnapGene software was used to integrate the adapted DNA sequence to pET-28a (+) vector, between the SacI and NheI restriction sites (Fig 10).

### mRNA prediction of the designed vaccine

**Fig 11:**
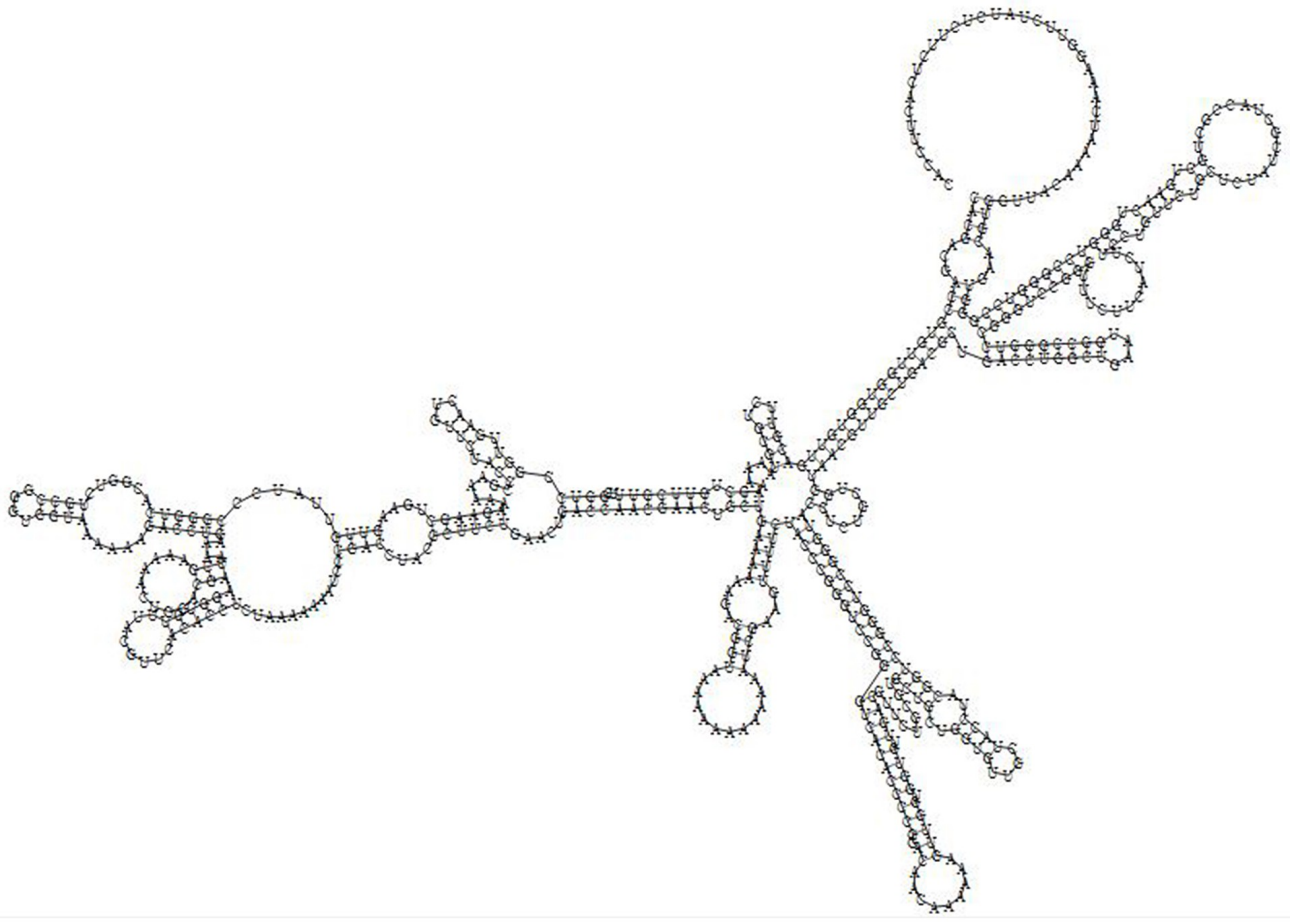
Prediction of mRNA secondary structure of multi-epitope vaccine by RNAfold server.

The secondary structure of the vaccine mRNA sequence was predicted by the RNAfold server with a MFE score of -163.70 kcal/mol. The prediction of the secondary structure of the multi-epitope vaccine mRNA is shown in Fig 11. This finding is in consistent with similar studies [68–72], which totally proposed that present vaccine may have stable mRNA structure following entry, transcript and expression in host.

## Discussion

β-lactam antibiotics are among the most widely used antimicrobials, and increased resistance to these drugs due to their widespread use is a public health concern. Enzymatic hydrolyzing of β-lactam ring is one of the most important mechanisms for resistance to β-lactam antibiotic among pathogenic bacteria [2]. The spectrum of activity against different classes of β-lactam varies between enzymes. Carbapenems such as MBLs, which hydrolyze all classes of β-lactams, except monobactams have attracted increasing attention due to the resistance to most β-lactam classes and the increasing frequency of isolated from patients [2, 73].

Acquired MBLs have been reported in several major gram-negative pathogens, including members of *Enterobacteriaceae* and *Pseudomonas* and *Acinetobacter*, which makes MBL-producing microorganisms as a serious public health concern [11, 74, 75]. The effectiveness of common antibiotics against these bacteria has decreased. Therefore, the production of vaccines against VIMs and IMPs variants as an alternative treatment strategy can play an important role in its prevention and treatment.

According to studies, immunization using β-lactamase enzymes produces neutralizing antibodies against the relevant β-lactamase enzymes and since antibodies can penetrate into the biofilm, in co-administration with antibiotics causes significant improvement of infections by resistant bacteria [76, 77]. On the basis of this fact, identification of β-lactamase epitopes and multi-epitope peptides as a potential antigens for immunization instead of the whole protein could be useful [78].

The location of MBLs (IMP and VIM) in the cell suggests that they do not appear to be good targets for inactivation in the vaccine approach, although this is under controversy among different studies. According to González et al study, these controversies may decline due to the fact that different localization sites of enzymes in the cell would determine being as membrane anchoring or soluble protein. Moreover, NDM-producing clinical strains secrete outer membrane vesicles (OMVs) containing NDM-1. They also showed OMVs containing NDM enzyme, provide an excellent potential target for new antibacterial strategy [21].

Based on the results of Fathollahi et al study, effective epitopes for vaccine design against NDM-producing pathogens have been reported [22]. Moreover, CIOFU et al believed that periplasmic beta-lactamase enzymes were packaged in MVs and released into the extracellular space [79]. In addition, Rumbo et al showed OXA-24 carbapenemases gene and related proteins were also carried by MVs [80]. It has been shown NDM has a LSGC amino acid sequence near to the peptide signal which is a marker for membrane-binding or solubility in the periplasm. Cys residue serve as lipidation site [81]. Many studies have shown that MBLs (IMP, VIM, and NDM) are present as soluble proteins in cells [12, 19–21].

Vaccines are an important tools in public health and play an important role in controlling infectious diseases, but the process of developing and producing them is costly and sometimes it takes years to get a suitable vaccine against a particular pathogen [69]. For this reason, scientists today are working on subunit vaccines using in silico approaches. It has also been shown that immunoinformatics methods are useful in predicting B cell and T cell epitope peptide antigens for the development of peptide vaccines [82].

Using the immunoinformatics method, we were able to select a total of 7 B cell and 4 HTL epitopes from both VIM and IMP proteins. Based on the scores and rankings of the epitopes in the results, we have 7 B cell epitopes, 111–122 aa (HDDRVGGVDVLR), 157–168 aa (AVRFGPVELFYP), 222–235 aa (EAEVVIPGHGLPGG), 23–38 aa (DLKIEKLDEGVYVHTS), 103–120 aa (IPTYASELTNELLKKDGK), 136–149 aa (KIEVFYPGPGHTPD), 139–150 aa (VFYPGPGHTPDN) and 4 CD4^+^ T cell epitopes, 115–129 aa (VGGVDVLRAAGVATY), 197–211 aa (TSAGNVADADLAEWP), 8–22 aa (FFIFLFCSIATAAEL) and 76–90 aa (ERGYKIKGSISSHFH). These epitopes were the most promising in terms of immunological characteristics and were selected for the final vaccine. Epitope vaccine candidates are also 100% conserved in both predicted T and B cell epitopes.

The vaccine fragments were joined by KK and GPGPG linkers. GPGPG binders stimulate the T-helper response as well as antibody epitopes. Finally, the candidate vaccine sequence is 174 amino acids long, which is suitable for such a vaccine. Multi-epitope vaccines can activate both humoral and cellular immune responses and thus are considered a better alternative to monovalent vaccines. Therefore, in the present study, we have attempted to design a new multi-epitope vaccine with knowledge of structural features through immunoinformatic methods to contribute to allergenic and immunogenicity properties.

Assessment of different physicochemical properties is essential to determine the safety and efficacy of multi-epitope vaccines [83]. The theoretical pI of our designed vaccine is alkaline (pI> 7) due to the presence of essential amino acids such as lysine and arginine, which are involved in protein activity and membrane voltage detection [84]. The aliphatic index is more than 70, which indicates that the vaccine is heat-stable in nature, because the higher the aliphatic index of a protein, the greater its thermal stability [45]. On the other hand, the GRAVY protein value of the designed vaccine was predicted to be negative (-0.370), indicating the fact that the vaccine is hydrophilic in nature. Another vital feature of any recombinant vaccine is its solubility [85]. For this reason, the solubility of our candidate vaccine was predicted to be 0.679, which indicates good solubility. The construct vaccine was found to be antigenicity, non-allergenicity, and non-toxic. These properties of the vaccine may elicit a strong immune response and also not cause an allergic reaction in the body.

The initial tertiary structure of the vaccine was predicted using the RaptorX server, and model 1 with a RMSD of 5.7610 Å was selected, indicating that the 3D structure vaccine was of good quality. Predicting the structure of the designed vaccine needs to be refinement and validation. For validation, about 83.2% of the amino acid residues in the region were optimal for Ramachandran diagram analysis by the PROCHECK server. The ERRAT tool, on the other hand, predicted a good quality vaccine structure with an overall quality score of 91,156, as the accepted range of the overall quality score produced by ERRAT for a high quality model is over 50 [52].

Molecular imaging is recognized as a key tool in structural molecular biology and computer-aided drug design. The goal of ligand-protein docking is to predict the predominant binding mode(s) of a ligand with a protein of known 3D structure [55]. To assess the interactions between TLR4 and the designed vaccine, docking was performed, and for the stability of docking complexes, MD simulations were applied. Subsequently, the best docked model was chosen. The docking score indicated that there was a significant interaction between the vaccine and the innate immune receptor, thus the vaccine could activate TLR and thus lead to increased immune responses. In summary, MD simulation findings confirm that the designed vaccine molecule can interact favorably with the TLR4 protein.

To ensure the efficient expression of the vaccine in *E*. *coli* host, codon optimization of the vaccine peptide was performed. The results obtained in JCat with CAI (1.0) and GC content (52%) were satisfactory because criteria such as determining CAI above 0.80 and GC content at 30–70% are considered as good scores [69].

Finally, predicting the stability of the secondary structure of the mRNA vaccine with the help of the RNAfold server produced a negative and lower MFE, so it can be proposed that the anticipated vaccine could be very stable after transcription in vivo. Mugunthan et al [72] study result was more similar to our observation which the MFE was reported as ΔG = −156.50 kcal/mol (present study result~ -163.70 kcal/mol). This finding is also consistent with similar studies [68–72, 86] which totally proposed that present vaccine may have stable mRNA structure following entry, transcript and expression in host.

## Conclusion

The rapid spread of acquired MBLs among gram negative pathogens is becoming a global concern. Although the efforts are focused only on developing new antibiotics, one of the best approaches to suppress antibiotic resistance is eliciting immune reactions against antibiotic resistant elements. In this study, immunoinformatics tools were used to construct a multi-epitope vaccine against MBLs that could trigger strong immune responses. The designed vaccine has a very good coverage against VIM and IMP MBLs in different populations with different genetic characteristics. However, in vitro and animal model studies are needed to demonstrate the efficacy of the designed vaccine.

## Supporting information

S1 File (XLSX)
